# Preferred Sources of Health Information in Persons With Multiple Sclerosis: Degree of Trust and Information Sought

**DOI:** 10.2196/jmir.2466

**Published:** 2013-04-30

**Authors:** Ruth Ann Marrie, Amber R Salter, Tuula Tyry, Robert J Fox, Gary R Cutter

**Affiliations:** ^1^University of ManitobaDepartments of Internal Medicine & Community Health SciencesWinnipeg, MBCanada; ^2^University of Alabama at BirminghamDepartment of BiostatisticsBirmingham, ALUnited States; ^3^Barrow Neurological InstituteDivision of NeurologyPhoenix, AZUnited States; ^4^Neurological InstituteMellen Center for MSCleveland ClinicCleveland, OHUnited States

**Keywords:** multiple sclerosis, Internet, social media, trust, health information

## Abstract

**Background:**

Effective health communication is important for informed decision-making, yet little is known about the range of information sources used by persons with multiple sclerosis (MS), the perceived trust in those information sources, or how this might vary according to patient characteristics.

**Objective:**

We aimed to investigate the sources of health information used by persons with MS, their preferences for the source of health information, and levels of trust in those information sources. We also aimed to evaluate how these findings varied according to participant characteristics.

**Methods:**

In 2011, participants in the North American Research Committee on Multiple Sclerosis (NARCOMS) Registry were asked about their sources of health information using selected questions adapted from the 2007 Health Information National Trends (HINTS) survey.

**Results:**

Of 12,974 eligible participants, 66.18% (8586/12,974) completed the questionnaire. Mass media sources, rather than interpersonal information sources, were the first sources used by 83.22% (5953/7153) of participants for general health topics and by 68.31% (5026/7357) of participants for MS concerns. Specifically, the Internet was the first source of health information for general health issues (5332/7267, 73.40%) and MS (4369/7376, 59.23%). In a logistic regression model, younger age, less disability, and higher annual income were independently associated with increased odds of use of mass media rather than interpersonal sources of information first. The most trusted information source was a physician, with 97.94% (8318/8493) reporting that they trusted a physician some or a lot. Information sought included treatment for MS (4470/5663, 78.93%), general information about MS (3378/5405, 62.50%), paying for medical care (1096/4282, 25.59%), where to get medical care (787/4282, 18.38%), and supports for coping with MS (2775/5031, 55.16%). Nearly 40% (2998/7521) of participants had concerns about the quality of the information they gathered.

**Conclusions:**

Although physicians remain the most trusted source of health information for people with MS, the Internet is the first source of health information for most of them. This has important implications for the dissemination of health information.

## Introduction

When seeking health information, patients use interpersonal sources including peers, and mass media sources such as the Internet. Preferred sources of health information vary by age, ethnicity, socioeconomic status and type of chronic condition [1-5]. Use of the Internet as a health information source, for example, varies from 35% in Canadians with spinal cord injury [2], to 62% in rheumatoid arthritis patients from New Jersey, United States, participating in a commercial health plan [3], to 63-82% in people with multiple sclerosis (MS) from regions in the United States and Israel [4,5].

Effective health communication is important for informed decision-making, yet little is known about the information sources used by people with MS, the perceived trust in those sources, or how this might vary by patient characteristics. Before their first visit at an MS clinic, 82% of people with MS from Ohio, United States, gathered information from the Internet [4]. Another study suggested that more disabled people with MS preferred interaction with health care providers over seeking information electronically; however, that study focused on Internet use in a clinic population and did not evaluate the breadth of information sources used [5].

We aimed to investigate the sources of health information used by people with MS, their preferences for the source of health information, and levels of trust in those information sources. We also aimed to evaluate how these findings varied according to participant characteristics.

## Methods

### North American Research Committee on Multiple Sclerosis Registry

The Consortium of MS Centers developed the North American Research Committee on Multiple Sclerosis (NARCOMS) Registry as a voluntary self-report registry for people with MS [6]. NARCOMS participants agree to the use of their de-identified data for research purposes, and the Registry is approved by the Institutional Review Board at the University of Alabama at Birmingham. Diagnoses of MS were validated in a randomly selected sample of participants [7]. Participants enrolled by mailing in a questionnaire or by completing a questionnaire online [6]. Thereafter, they completed surveys semi-annually, on paper, or online. At enrollment and on the semi-annual surveys, participants provided sociodemographic and clinical information. Disability status was reported using Patient Determined Disease Steps (PDDS) [8], a validated measure that correlates well with a physician-scored Expanded Disability Status Scale (EDSS) [8]. It is scored ordinally from 0 to 8, where a score of 0 approximates an EDSS score of 0, a score of 3 represents early gait disability without needing an assistive device and approximates an EDSS score of 4.0 to 4.5; and scores of 4, 5, and 6 represent EDSS scores of 6 to 6.5.

### Questionnaire

In 2011, participants were asked about sources of health information using questions adapted from the 2007 Health Information National Trends (HINTS) survey [9]. HINTS was developed by the National Cancer Institute to evaluate changing trends in health communication, to assess access to and use of cancer information, and to evaluate perception of cancer risk and health behaviors [9]. The first section of the survey queried information seeking about health topics during the respondents’ most recent search, the second inquired about information seeking about cancer, and the third focused on Internet use. Collectively, this survey captured information seeking from interpersonal and mass media sources. We substituted MS for cancer in these sections, and substituted MS organizations (such as the National Multiple Sclerosis Society [NMSS] and Consortium of Multiple Sclerosis Centers for cancer organizations. The adapted questions can be reviewed in Multimedia Appendix 1.

### Statistical Analysis

Because of variability in the availability of health care information sources and in health care systems worldwide, we restricted our analysis to NARCOMS participants living in the United States. Missing responses were not imputed. Note that some participants did not respond to all questions, and some data were missing for non-responders; therefore we report the number of individuals responding to each question throughout. We summarized categorical variables using frequency (percent) and continuous variables using mean (standard deviation) or median (interquartile range) as appropriate.

Use of information sources was tabulated by individual source where only one choice could be selected, and categorized as interpersonal (family, provider, friend, patient advocacy organization, [10]) or mass media sources (books, newspapers, brochures, library, magazines, Internet). Using logistic regression we examined demographic and clinical factors associated with use of mass media versus interpersonal sources of information, factors associated with Internet use (yes vs no), the type of information (yes vs no) sought, including information sought by more than 50% of participants and regarding access to care. We identified health information sources reported by ≥5% of participants as their first information source, and examined the association between sociodemographic and clinical characteristics, and level of trust for those information sources using logistic regression models. For each health information source, trust was dichotomized as more (a lot or some) versus less (a little or not at all) [11].

The independent variables considered for each regression model are described below. Race was categorized as white (reference group), and non-white. Education was included as indicator variables for high school diploma or less (reference group), Associate’s Degree or Technical Degree, Bachelor’s Degree, and post-graduate degree. Annual household income was included as indicator variables for <$15,000 (reference group), $15,000-29,999, $30,000-49,999, $50,000-100,000, >$100,000, or declined to answer. Insurance status was included as indicator variables for private, public only (reference group), or none. Region of residence was included as indicator variables for West (reference group), Midwest, South, and East as defined by the US Census bureau. Age was categorized as 18-34, 35-49, 50-59, and ≥60 (reference group) years. Disease duration was categorized into quartiles, thus 0-16, 17-24, 25-33 and ≥34 years. Using PDDS, participants were classified as having mild (0-2), moderate (3-4), or severe (5-8, reference group) disability [12]. We also included a variable indicating whether the questionnaire was completed online or on paper.

Assumptions of models were tested using standard methods [13]. For each model, we reported a c-statistic as a measure of discriminating ability (estimate of area under the curve) and the Hosmer Lemeshow test as a measure of goodness of fit. Analyses were performed using SAS V9.2.

## Results

### Respondents

Of 12,974 eligible participants, 8586/12,974 (66.18%) responded. The demographic and clinical characteristics of the responders are summarized in Table 1. As compared to responders, non-responders were more likely to be women (80.90%, 3542/4378 vs 77.56%, 6650/8574, *P*<.0001), and of lower socioeconomic status (annual income at enrollment <$15,000; 458/4219, 10.86% vs 795/8176, 9.72%, *P*=.047). Non-responders had a lower age of symptom onset (mean 30.0, SD 9.7 years) than responders (mean 30.9, SD 10.0 years), and a lower age of diagnosis (mean 37.7, SD 9.8 vs mean 38.5, SD 9.7, both *P*<.0001). Although these differences were statistically significant, the differences in the absolute values were so small that they were unlikely to be clinically significant. Responders and non-responders did not differ with respect to the severity of disability at enrollment.

### Information Sources

Eighty-nine percent of respondents (7512/8439) reported ever seeking information about health or medical topics from any source. Participants who completed the survey online were more likely to answer this question affirmatively (5291/5635, 93.90%) than those who did not (2221/2951, 75.26%, *P*<.0001). Similarly, 88.13% (7459/8464) reported that they had ever sought information about MS specifically, and such information-seeking behavior was greater among participants who completed the survey online (5157/5610, 91.92%) versus paper (2302/2854, 80.66%, *P*<.0001)

Table 2 shows the first source that participants went to for information about general health or medical topics, for the most recent time that they sought information. The Internet was the most common choice, reported by 73.37% (5332/7267) of participants who responded to the question and correctly selected only one choice. The second choice was health care providers (8.48%, 616/7267), followed by the NMSS (400/7267, 5.50%). The Internet was a less common first source of information regarding MS specifically, being reported by 4369/7267 (59.23%) of participants. Health care providers remained the second choice (1127/7376, 15.28%) and the NMSS remained the third choice (962/7376, 13.04%).

For further analysis, information sources were categorized as interpersonal or mass media. Mass media was the first information source used by 83.22% (5953/7153) of participants for general health topics and by 68.31% (5026/7357) of participants for MS concerns. On univariate analysis, characteristics associated with greater mass media use were female sex (*P*=.0048), younger age (*P*<.0001), greater than a high school education (*P*<.0001), higher annual income (*P*<.0001), survey completion online (*P*<.0001), and less disability (*P*<.0001). In a logistic regression model, younger age, less disability, and higher annual income were independently associated with increased odds of use of mass media rather than interpersonal sources of information first (Table 3).

### Health Information Sought

Participants sought a broad range of information regarding MS, including general aspects of MS, treatment, access to health care, and support for coping with MS (Table 4). Using multivariable logistic regression, we evaluated the association between participant characteristics and the types of information sought by more than 50% of participants (Multimedia Appendix 2). Consistently, higher socioeconomic status was associated with lower odds of searching for information. Lower levels of disability were associated with increased odds of searching for information about symptoms, while longer disease duration was associated with decreased odds of searching for information about coping and symptoms.

Information regarding access to care was also sought frequently on topics including paying for medical care (1096/4282, 25.59%), insurance (1008/4393, 22.95%), and where to get medical care (787/4282, 18.38%). Using logistic regression, lower socioeconomic status was consistently associated with searching for information regarding access to care (Multimedia Appendix 3).

### Internet Use

Most participants had accessed the Internet or used email (7292/8469, 86.10%), with more than 60% (4736) indicating that they accessed the Internet several times a day. Participants who completed the questionnaire online used the Internet more frequently than those who completed the questionnaire on paper (linear trend *P*<.0001). Using logistic regression, younger age, higher educational level, higher annual income, and milder disability were independently associated with increased odds of Internet use (Multimedia Appendix 4). Non-white race and public rather than private health insurance was associated with decreased odds of Internet use. Internet use varied somewhat across regions, being lower in the East and Midwest regions than the West.

Participants reported conducting several other activities online including social networking (4606/7528, 61.18%), seeking a health care provider (2595/7516, 34.53%), buying medications or vitamins (2583/7537, 34.27%), communicating with a physician (2408/7536, 31.95%), seeking advice regarding diet, weight, or physical activity (2401/7522, 31.92%), tracking personal health information such as test results or medical appointments (2159/5366, 28.69%), downloading information to a device (1971/7516, 26.21%), using online support groups for people with MS (1557/7526, 20.69%) and blogging (584/7515, 7.77%).

### Satisfaction and Trust

A substantial proportion of respondents had concerns regarding their search for information. Specifically, 2131/7556 (28.20%) felt that it took a lot of effort to get the information required, while 2120/7531 (28.15%) felt frustrated during their search, 2998/7521 (39.86%) were concerned about the quality of information gathered, and 1590/7533 (21.11%) thought that the information obtained was hard to understand.

**Figure 1:**
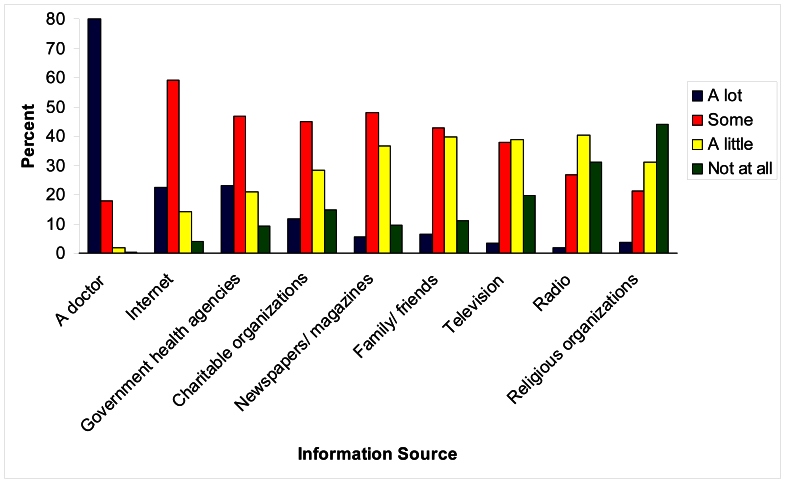
Degree of trust in various information sources.

The degree of trust varied across information sources. The most trusted source of information was a physician, with 97.94% (8318/8493) reporting that they trusted a physician some or a lot (Figure 1). Table 5 shows the results of logistic regression analyses for the association of trust (more vs less) and sociodemographic and clinical characteristics for the three most commonly used information sources for MS-related information (physicians, the Internet, and patient advocacy groups). Although some associations varied according to the information source, general patterns emerged. Higher levels of education and income were associated with greater trust in the three sources, as was mild rather than severe disability. Although age was not associated with trust in doctors, younger age was associated with increased trust in the Internet and patient advocacy groups.

**Table 1:** Demographic and clinical characteristics of responders to the NARCOMS Fall 2011 questionnaire.

Characteristic		n/N (%) or mean (SD)
**Sex**		
	Female	6649/8573 (77.56)
	Male	1924/8573 (22.44)
**Race**		
	White	7610/7972 (95.46)
	Non-White	362/7972 (4.54)
**Education**		
	High school diploma or less	2777/8505 (32.65)
	Associate's or Technical degree	1457/8505 (17.13)
	Bachelor's degree	2392/8505 (28.12)
	Post-graduate degree	1879/8505 (22.09)
**Annual income**		
	<$15,000	706/8362 (8.44)
	$15,000-29,999	1249/8362 (14.94)
	$30,000-49,999	1347/8362 (16.11)
	$50,000-100,000	2020/8362 (24.16)
	>$100,000	1194/8362 (14.28)
	I do not wish to answer	1846/8362 (22.08)
**Health insurance**		
	Private	5612/8384 (66.94)
	Public only	2561/8384 (30.55)
	None	211/8384 (2.52)
**Region of residence**		
	East	1981/8579 (23.09)
	Midwest	2180/8579 (25.41)
	South	2237/8579 (26.08)
	West	2181/8579 (25.42)
**Current age (years), mean (SD)**		
		56.6 (10.5)
**Age of symptom onset (years), mean (SD)**		
		30.9 (10.0)
**Age of diagnosis (years), mean (SD)**		
		38.5 (9.7)
**Patient determined disease steps (categorized)**		
	Mild (0-2)	3009/8507 (35.37)
	Moderate (3-4)	2216/8507 (26.05)
	Severe (5-8)	3282/8507 (38.58)
**Questionnaire administration**		
	Online	5635/8586 (65.63)
	Paper	2951/8586 (34.37)

**Table 2:** The first source for health information, the last time it was sought (N=7376).

Source of health information	General health n (%)	Multiple Sclerosis n (%)
Internet	5332 (73.40)	4369 (59.23)
Doctor or health care provider	616 (8.48)	1127 (15.28)
National Multiple Sclerosis Society	400 (5.50)	962 (13.04)
Books	267 (3.67)	175 (2.37)
Magazines	141 (1.94)	174 (2.36)
Other	115 (1.58)	116 (1.57)
Brochures, pamphlets, etc	104 (1.43)	142 (1.93)
Family	78 (1.07)	44 (0.60)
Consortium of MS Centers	39 (0.54)	84 (1.14)
Telephone information number	50 (0.69)	43 (0.58)
Newspapers	36 (0.50)	22 (0.30)
Complementary or alternative practitioner	35 (0.48)	35 (0.47)
Friend/co-worker	32 (0.44)	41 (0.56)
Library	22 (0.30)	42 (0.57)

**Table 3:** Characteristics associated with using mass media versus interpersonal information sources as the first source of health information (n=6348).^a^

Characteristic		Odds Ratio	95% CI
**Age group, years**			
	18-34	1.38	0.91, 2.10
	35-49	2.01	1.65, 2.44
	50-59	1.58	1.37, 1.83
	≥60 (Reference^b^)	1.0	
**Annual income**			
	<$15,000 (Reference^b^)	1.0	
	$15,000-29,999	1.44	1.12, 1.86
	$30,000-49,999	1.79	1.38, 2.31
	$50,000-100,000	2.05	1.60, 2.62
	>$100,000	2.65	1.98, 3.54
	Declined to answer	1.57	1.23, 2.00
**Disability**			
	Mild	1.44	1.12, 1.87
	Moderate	1.11	1.38, 2.31
	Severe (Reference^b^)	1.0	

^a^c-statistic = 0.63; HLGOF χ^2^_8_= 13.5, *P*=.09

^b^ reference group in the regression model

**Table 4:** Type of health information sought about MS at the time of the most recent search.

Type of health information sought	n/N (%)
Treatment for MS	4470/5663 (78.93)
General information about MS	3378/5405 (62.50)
Symptoms of MS	2810/5032 (55.84)
Coping with MS	2775/5031 (55.16)
Complementary and alternative therapies	2222/4808 (46.21)
MS organizations	1962/4616 (42.50)
Cause of MS	1914/4742 (40.36)
Prognosis	1701/4610 (36.90)
Diagnosis of MS	812/4253 (19.09)
Other information	976/3755 (25.99)

**Table 5:** Demographic and clinical characteristics associated with trust (“some” or “a lot”) versus lack of trust in sources of health information.

Characteristic		Information Source		
		Physician^a^ (Nn=7559)	Internet^b^ (N=7380)	Patient advocacy group^c^ (N=7414)
		OR (95% CI)	OR (95% CI)	OR (95% CI)
**Sex**				
	Female	1.0	1.0	1.0
	Male	1.29 (0.84, 1.98)	0.72 (0.62, 0.82)	1.05 (0.94, 1.18)
**Age group, years**				
	18-34	0.54 (0.21, 1.40)	1.04 (0.68, 1.59)	2.54 (1.75, 3.67)
	35-49	1.24 (0.75, 2.05)	1.42 (1.19, 1.68)	1.55 (1.35, 1.77)
	50-59	1.02 (0.70, 1.48)	1.50 (1.30, 1.73)	1.40 (1.26, 1.56)
	≥60 (Reference^d^)	1.0	1.0	1.0
**Race**				
	White	1.0	1.0	1.0
	Other	1.00 (0.46, 2.18)	1.05 (0.78, 1.40)	0.99 (0.79, 1.25)
**Education**				
	High school or less	1.0	1.0	1.0
	Associate’s/Technical degree	1.50 (0.92, 2.44)	1.31 (1.10, 1.57)	1.08 (0.94, 1.25)
	Bachelor’s degree	1.88 (1.15, 3.00)	1.37 (1.17, 1.61)	1.28 (1.13, 1.45)
	Graduate degree	1.38 (0.86, 2.22)	1.30 (1.10, 1.55)	1.25 (1.13, 1.45)
**Annual income**				
	<$15,000	1.0	1.0	1.0
	$15,000-29,999 (Reference^d^)	1.43 (0.85, 2.42)	1.41 (1.11, 1.79)	1.04 (0.85, 1.28)
	$30,000-49,999	3.45 (1.75, 6.78)	1.68 (1.31, 2.16)	1.16 (0.94, 1.43)
	$50,000-100,000	2.80 (1.51, 5.19)	1.97 (1.53, 2.52)	1.33 (1.08, 1.63)
	>$100,000	2.00 (0.99, 4.03)	2.17 (1.62, 2.89)	1.39 (1.10, 1.74)
	Declined to answer	2.02 (1.17, 3.51)	1.42 (1.12, 1.79)	0.89 (0.72, 1.08)
**Insurance**				
	Public only	1.0	1.0	1.0
	Private	1.11 (0.76, 1.63)	1.04 (0.69, 1.57)	1.11 (0.81, 1.53)
	None	1.65 (0.51, 5.38)	0.98 (0.84, 1.13)	1.10 (0.98, 1.24)
**Region**				
	West	1.0	1.0	1.0
	East	1.08 (0.84, 1.74)	0.88 (0.74, 0.82)	1.04 (0.94, 1.18)
	Midwest	1.17 (0.67, 1.87)	0.84 (0.71, 1.04)	1.06 (0.91, 1.21)
	South	0.98 (0.63, 1.54)	1.07 (0.90, 1.27)	1.23 (0.93, 1.40)
**Disability**				
	Mild	1.34 (0.87, 2.25)	1.21 (1.04, 1.41)	1.28 (1.14, 1.44)
	Moderate	1.09 (0.72, 1.75)	1.07 (0.92, 1.25)	1.08 (0.96, 1.22)
	Severe (Reference^d^)	1.0	1.0	1.0

^a^c-statistic = 0.66, Hosmer Lemeshow Goodness of Fit (HLGOF) χ^2^_8_ = 7.16, *P*=.52

^b^c-statistic = 0.61, HLGOF χ^2^_8_ = 8.45, *P*=.39

^c^c-statistic = 0.61, HLGOF χ^2^_8_ = 15.3, *P*=.054

^d^reference group in the regression model

## Discussion

### Principal Results

We investigated information sources used by people with MS, satisfaction and trust in those information sources, and the type of information sought about MS. In 2011, more than 85% of our participants reported Internet access. Four years earlier, 69% of American adults who responded to the HINTS survey, which was adapted for our study, reported having access to the Internet [14]. With a frequency of more than 70%, the Internet was the most common first source of general health information reported by study participants at the time of their most recent search, reported 8-fold more often than health care providers, and 13-fold more often than patient advocacy organizations. Although the Internet was a slightly less frequent first choice of information for MS, it was still frequently used (59.23%, 4369/7376). Similarly, one clinic-based study of 96 people with MS from Israel found that 63% used the Internet for MS related searches [5]. In the general population, the Internet is also the first source of cancer information, and this is rising, from just under 50% in 2002-2003 to over 55% in 2008 [15]. In the general population it is also notable that the Internet is less often the first source of information for cancer, than for general health information, similar to our findings for MS [11]. The reasons for these differences are uncertain but the greater complexity of disease-specific information might drive patients to seek more information from their health care providers. Prior studies of the MS population have found that those who used the Internet were younger and more educated than those who did not [5], as have studies of cancer populations [16]. Similarly, we found that higher levels of income, having private health insurance, and white race were associated with more Internet use. We did not evaluate whether this related to computer access, but this should be explored.

Although the Internet was the first source of information reported, 80.02% (6796/8493) of our participants reported a lot of trust in physicians while only 22.63% (1872/8271) reported a lot of trust in the Internet. This is similar to findings in the general population. In the 2008 HINTS survey, trust in physicians was higher with 80% of respondents from the general population reporting the highest trust in information from that source, and only 20% reporting a lot of trust in the Internet [15]. The Internet remained, however, the first information source for more than 55% of respondents.

We found that participants conducted multiple health-related activities online in addition to information seeking (Multimedia Appendix 5). In 2003, the first HINTS survey found that 3.9% of their respondents participated in an online support group and 9.1% bought medicine or supplements online [11]. In 2007, the HINTS survey found that 5% of their respondents participated in an online support group, 14.5% bought medicine online, and 23% used a social networking site [14]. In 2011, NARCOMS participants reported substantially higher use of the Internet for such activities, with 20% using online support groups, 34% buying medication or supplements online, and more than 60% participating in social networking. Although use of the Internet for health reasons is increasing in the general population, these findings suggest it may be higher among people with MS.

The range of information sought regarding MS by NARCOMS participants highlights the varied information needs of people with MS, some of which are likely to change over the disease course. In our study, 18-24% of participants sought information regarding topics related to access to care, including where to get care and how to pay for it. Such information was most often sought by people of lower socioeconomic status. This underscores the economic challenges associated with MS care. In a sample of 2156 people with MS, lower socioeconomic status including lower family income and lack of health insurance, was associated with a lower probability of receiving care from a neurologist [17]. Lower socioeconomic status was also associated with disparities in care for urinary symptoms [18] and mental comorbidity [19].

### Comparison With Prior Work

A mixed methods study of 61 people attending a first visit at an MS clinic found that 82% sought information on the Internet before that appointment [4], highlighting that information seeking often begins even prior to a confirmed diagnosis. These individuals reported doing Internet searches to gather background information, save time during appointments, to verify physician competency, and to find an MS physician and to obtain social support. In an Australian study of 23 people newly diagnosed with MS, most wanted information from the MS Society and MS specialist nurses. They were happy with the amount of information received from those sources, but wanted more information than they currently received from neurologists, family physicians, and education sessions [20]. Thus people with MS frequently seek many types of information from a range of sources, but remain dissatisfied with the amount of information obtained from some sources. This is consistent with the disparity between the first choice of information source and the most trusted information source noted in the present study. Further, despite the readily available information from sources such as the Internet, 39.86% (2998/7521) of participants in our study had concerns regarding information quality, and 21.11% (1590/7553) found the information difficult to comprehend. Participants of lower socioeconomic status reported more difficulty finding and understanding information.

Collectively, these findings have important implications for dissemination of information to people with MS and their families. Health care providers should be aware that their patients are likely to gather considerable information from the Internet, and typically before their patients obtain information from their providers. Further, although patients trust the information obtained from their physicians, they likely want more information from them, and in a more timely fashion. Traditional information sources such as newspapers, television, and radio are likely to be ineffective methods of communication as they are either not used, not trusted, or both. The Internet, including social media, provides a means for rapid dissemination of health information by health care providers, which can be readily updated. However, it is critical that this information be provided in a way that is readily accessible and comprehensible to people of all socioeconomic backgrounds.

### Limitations

This study has limitations. Our response rate of 66.18% (8586/12,974) was lower than desired, but this may be an underestimate of the true response rate since we could not determine how many participants actually received the questionnaire as changes in home or email addresses and deaths are often identified only long after a participant has failed to respond. The NARCOMS Registry is a voluntary registry, therefore it does not fully represent the MS population in the United States, and non-responders had a lower socioeconomic status than responders. However, the NARCOMS population is large and sociodemographically diverse with characteristics similar to those reported for other MS populations [21]. A key strength of this study was that participants included those who responded online and by mail, avoiding the limitations of other studies about Internet use which have been limited to online only completion. Further, this was the largest study related to this issue that we know of.

### Conclusions

Our work highlights the main sources of health information used by people with MS, and thus has implications for the dissemination of health information, keeping in mind the discordance between the most readily accessible source, the Internet and the most trusted resource. The rise in the use of social networking, and platforms facilitating direct exchange of personal health information between patients [22,23] are dramatically changing patterns of health communication.

## Multimedia Appendix 1

Questions adapted from the Health Information Trends Survey (HINTS).

## Multimedia Appendix 2

Demographic and clinical characteristics associated with seeking general information regarding MS, using binary logistic regression.

## Multimedia Appendix 3

Demographic and clinical characteristics associated with seeking information regarding access to care using binary logistic regression.

## Multimedia Appendix 4

Demographic and clinical characteristics associated with internet use using binary logistic regression (n=7553).

## Multimedia Appendix 5

Health-related online activities reported by the NARCOMS population and respondents to the HINTS surveys.

## Abbreviations

EDSS: Expanded Disability Status Scale
HINTS: Health Information National Trends
MS: multiple sclerosis
NARCOMS: North American Research Committee on Multiple Sclerosis registry
NMSS: National Multiple Sclerosis Society
PDDS: Patient Determined Disease Steps
