# Supplementary material for: Preferred Sources of Health Information in Persons With Multiple Sclerosis: Degree of Trust and Information Sought
Source: J Med Internet Res. 2013 Apr 30;15(4):e67. doi: 10.2196/jmir.2466 (PMC3650929; doi:10.2196/jmir.2466)
Supplement: Supplementary file 1 [file jmir_v15i4e67_app1.pdf]

Multimedia Appendix 1. Questions adapted from the Health Information Trends Survey (HINTS).

Several questions from the HINTS were adapted to focus on multiple sclerosis (MS) rather than cancer. These are presented below. Question numbers correspond to those used in the full questionnaire.

A. Seeking Information about Health

29. Have you ever looked for information about health or medical topics from any source?

☐ 1. Yes ☐ 2. No **(Go to Question 34)**

30. The most recent time you looked for information about health or medical topics, where did you go **first**? **Mark only one.**

☐ 1. Books ☐ 6. Brochures, pamphlets, etc. ☐ 10. Friend/co-worker
☐ 2. Family ☐ 7. (National) Multiple Sclerosis Society ☐ 11.Consortium of MS Centers
☐ 3. Internet ☐ 8. Doctor or health care provider ☐ 12. Library
☐ 4. Newspapers ☐ 9. Telephone information number ☐ 13. Magazines
☐ 5. Complementary, alternative, or unconventional practitioner ☐ 14. Other → *Please specify:*

____

31. Did you look or go anywhere else? **Mark all that apply.**

☐ 1. Books ☐ 6. Brochures, pamphlets, etc. ☐ 10. Friend/co-worker
☐ 2. Family ☐ 7. (National) Multiple Sclerosis Societ ☐ 11.Consortium of MS Centers
☐ 3. Internet ☐ 8. Doctor or health care provider ☐ 12. Library
☐ 4. Newspapers ☐ 9. Telephone information number ☐ 13. Magazines
☐ 5. Complementary, alternative, or unconventional practitioner ☐ 14. Other→ *Please specify:*

____

B. Seeking Information about MS

36. Have you ever looked for information about MS from any source?

☐ 1. Yes ☐ 2. No **(Go** [illegible]

37. Think about the most recent time you looked for information on MS.
About how long ago was that? **Write a number in only ONE blank below**

_______Days _______Weeks _______Months _______Years

38. What type of information were you looking for? **Mark all that apply.**

☐ 1. Information on MS in general
☐ 2. Causes of MS/Risk factors for MS
☐ 3. Symptoms of MS
☐ 4. Paying for medical care
☐ 5. Where to get medical care
☐ 6. Information on complementary, alternative, or unconventional treatments
☐ 7. Diagnosis of MS
☐ 8. MS organizations
☐ 9. Coping with MS/Dealing with MS
☐ 10. Treatment for MS
☐ 11. Insurance
☐ 12. Prognosis of MS
☐ 13. Other → *Please specify:*__________________

39. The most recent time you looked for MS information, where did you go **first**?
Mark only one.

☐ 1. Books
☐ 2. Family
☐ 3. Internet
☐ 4. Newspapers
☐ 5. Complementary, alternative, or unconventional practitioner
☐ 6. Brochures, pamphlets, etc.
☐ 7. (National) Multiple Sclerosis Society
☐ 8. Doctor or health care provider
☐ 9. Telephone information number
☐ 10. Friend/co-worker
☐ 11.Consortium of MS Centers
☐ 12. Library
☐ 13. Magazines
☐ 14. Other→ *Please specify*
____

D. Nutrition and Physical Activity

51. As far as you know, which of the following best describes the effect of physical activity or exercise on the chances of **getting a MS relapse**?
☐ 1. Physical activity increases chances of a relapse
☐ 2. Physical activity decreases chances of a relapse
☐ 3. Physical activity makes no difference

52. As far as you know, which of the following best describes the effect of physical activity or exercise on the chances of **worsening symptoms of MS?**
☐ 1. Physical activity increases chances of worsening symptoms
☐ 2. Physical activity decreases chances of worsening symptoms
☐ 3. Physical activity makes no difference

53. As far as you know, which of the following best describes the effect of physical activity or exercise on the chances of **MS progressing**?
☐ 1. Physical activity increases chances of MS progressing
☐ 2. Physical activity decreases chances of MS progressing
☐ 3. Physical activity makes no difference
