# Supplementary material for: Preferred Sources of Health Information in Persons With Multiple Sclerosis: Degree of Trust and Information Sought
Source: J Med Internet Res. 2013 Apr 30;15(4):e67. doi: 10.2196/jmir.2466 (PMC3650929; doi:10.2196/jmir.2466)
Supplement: Supplementary file 3 [file jmir_v15i4e67_app3.pdf]

Multimedia Appendix 3. Demographic and clinical characteristics associated with seeking information regarding access to care using binary logistic regression.

**Characteristic**		**Information Sought Regarding Access to Care**		
		**Insurance[a]**	**Pay Med[b]**	**Get Meds[c]**
		**OR (95% CI)**	**OR (95% CI)**	**OR (95% CI)**
Sex	Female		1.0	
	Male		0.75 (0.62, 0.91)	
Age group, years	18-34	1.0	1.0	
	35-49	1.62 (1.00, 2.60)	1.45 (0.89, 2.35)	
	50-59	0.82 (0.66, 1.02)	0.78 (0.63, 0.98)	
	≥60 (Reference)	0.97 (0.81, 1.16)	1.15 (0.96, 1.38)	
Education	High school or less		1.0	1.0
	Associate’s/ Technical degree		0.83 (0.67, 1.04)	0.89 (0.70, 1.13)
	Bachelor’s degree		0.75 (0.61, 0.91)	0.58 (0.46, 0.73)
	Graduate degree		0.64 (0.51, 0.80)	0.57 (0.46, 0.73)
Annual income	<$15,000 (Reference)	1.0	1.0	1.0
	$15,000-29,999	0.82 (0.59, 1.16)	1.10 (0.80, 1.51)	0.49 (0.34, 0.69)
	$30,000-49,999	0.67 (0.48, 0.94)	0.59 (0.43, 0.83)	0.39 (0.28, 0.56)
	$50,000-100,000	0.40 (0.28, 0.56)	0.35 (0.25, 0.48)	0.28 (0.20, 0.41)
	>$100,000	0.26 (0.18, 0.38)	0.23 (0.16, 0.34)	0.25 (0.17, 0.38)
	Declined to answer	0.47 (0.34, 0.67)	0.45 (0.32, 0.62)	0.37 (0.26, 0.52)
Insurance	Public only	1.0	1.0	1.0
	Private	1.08 (0.89, 1.30)	0.90 (0.75, 1.08)	0.96 (0.78, 1.18)
	None	2.72 (1.71, 4.30)	3.22 (2.03, 5.11)	2.25 (1.42, 3.57)
Region	West			1.0
	East			0.91 (0.71, 1.18)
	Midwest			0.82 (0.64, 1.05)
	South			1.29 (1.03, 1.62)
Disease duration, years	0-16			1.0
	17-24			0.78 (0.62, 0.99)
	25-33			0.97 (0.76, 1.23)
	≥34			1.27 (0.99, 1.61)

a – c-statistic = 0.62, HLGOF χ^2 = 2.70, *P* = .91; b – c-statistic = 0.69, HLGOF χ^2 = 8.42, *P* = .39; c – c-statistic = 0.66, HLGOF χ^2 = 4.28, *P* = .83
