# Supplementary material for: Preferred Sources of Health Information in Persons With Multiple Sclerosis: Degree of Trust and Information Sought
Source: J Med Internet Res. 2013 Apr 30;15(4):e67. doi: 10.2196/jmir.2466 (PMC3650929; doi:10.2196/jmir.2466)
Supplement: Supplementary file 4 [file jmir_v15i4e67_app4.pdf]

Multimedia Appendix 4. Demographic and clinical characteristics associated with internet use using binary logistic regression (n = 7553).

**Characteristic**		**OR**	**95% CI**
Sex	Female	1.0	
	Male	0.86	0.72, 1.02
Age group, years	18-34	41.2	5.67, 299.3
	35-49	8.10	5.89, 11.1
	50-59	3.12	2.64, 3.70
	≥60 (Reference)	1.0	
Race	White	1.0	
	Other	0.69	0.50, 0.96
Education	High school or less	1.0	
	Associate's/Technical degree	1.71	1.39, 2.09
	Bachelor's degree	3.14	2.55, 3.88
	Graduate degree	2.74	2.19, 3.43
Annual income	<$15,000 (Reference)	1.0	
	$15,000-29,999	1.94	1.51, 2.47
	$30,000-49,999	2.43	1.88, 3.16
	$50,000-100,000	4.02	3.04, 5.34
	>$100,000	6.49	4.21, 10.0
	Declined to answer	2.44	1.91, 3.13
Health Insurance	Public only	1.0	
	Private	0.59	0.39, 0.90
	None	1.39	1.19, 1.64
Region	West (Reference)	1.0	
	East	0.67	0.54, 0.82
	Midwest	0.73	0.59, 0.89
	South	1.01	0.81, 1.25
Disability	Mild	1.88	1.54, 2.29
	Moderate	1.63	1.35, 1.96
	Severe (Reference)	1.0	

c-statistic = 0.81, Hosmer Lemeshow Goodness of Fit $\chi^2 = 5.27$, $P = .73$
