# Supplementary material for: Preferred Sources of Health Information in Persons With Multiple Sclerosis: Degree of Trust and Information Sought
Source: J Med Internet Res. 2013 Apr 30;15(4):e67. doi: 10.2196/jmir.2466 (PMC3650929; doi:10.2196/jmir.2466)
Supplement: Supplementary file 5 [file jmir_v15i4e67_app5.pdf]

Multimedia Appendix 5. Health-related online activities reported by the NARCOMS population and respondents to the Health Information National Trends (HINTS) surveys.

**Population (Year)**	**Online Activity**					
	**Support group**	**Buy medications**	**Blogging**	**Social networking**	**Tracking personal health information**	**Downloading information to device (e.g. phone)**
HINTS (2003)	3.9%	9.1%	-	-	-	-
HINTS (2005)	3.9%	12.8%	-	-	-	-
HINTS (2007)	5.0%	14.5%	7.0%	22.9%	15.2%	22.3%
NARCOMS (2011)	20.7%	34.3%	7.8%	61.2%	28.7%	26.2%
